# Boosting health provider performance with non-financial incentives: A cluster-randomized controlled trial in Tanzania

**DOI:** 10.1371/journal.pone.0330989

**Published:** 2025-09-11

**Authors:** Calvin Chiu, Agatha Mnyippembe, Lila A. Sheira, Laura Packel, Emmanuel Katabaro, Werner Maokola, Jenny X. Liu

**Affiliations:** 1 Institute for Health and Aging, University of California San Francisco, San Francisco, California, United States of America; 2 Health For A Prosperous Nation, Dar es Salaam, Tanzania; 3 Ministry of Health/National AIDS Control Programme, Dodoma, Tanzania; University of Lahore - Raiwind Road Campus: The University of Lahore, PAKISTAN

## Abstract

**Background:**

Non-financial incentives are frequently used to improve performance among healthcare providers, capitalizing on mission-driven intrinsic pro-social motivation. However, the effectiveness of incentives varies across settings and may depend on whether they are provided privately or publicly. Using a cluster-randomized controlled trial (ClinicalTrials.gov: NCT05525533) among drug shopkeepers in Tanzania, we designed and evaluated the effectiveness of non-financial incentives in boosting provider performance.

**Methods:**

We developed a non-financial incentive that involved providing shopkeepers with monthly reports of aggregated customer feedback compiled from anonymous surveys from young women (15–24) to appeal to shopkeepers’ pro-social motivation for helping these customers. We randomized whether the feedback was provided privately (via a report) or publicly (displaying certificates of the customer feedback ratings). We estimated linear regression models on provider performance as measured by sales across different categories to young women from administrative point-of sales data over 12 months and estimated whether performance measures were correlated with shopkeepers’ pro-social motivation and concern for social image measured by surveys at Baseline.

**Results:**

Young women customers completed 9,108 anonymous surveys across 99 shops. Shops receiving non-financial incentives privately did not increase performance. However, shops receiving non-financial incentives publicly reported an increase in sales to young women customers (58%, 95% CI: 20%, 97%), most notably for sexual and reproductive health products (96%, 95% CI: 4%, 187%), specifically oral contraception (154%, 95% CI: 9%, 306%) and pregnancy tests (75%, 95% CI: 8%, 143%). Performance measures were correlated with concern for social image but not pro-social motivation at baseline.

**Conclusions:**

Publicly provided non-financial incentives increased performance among drug shopkeepers in Tanzania serving young women. Performance was strongest among those with higher concern for their social image at baseline, rather than those with stronger pro-social motivation. Future interventions using non-financial incentives to motivate healthcare providers should consider leveraging providers’ social image concerns to amplify the effectiveness of incentives.

## Introduction

Given the global shortage of healthcare providers, particularly in low and middle-income countries [1], health systems need to maximize the productivity of their scarce healthcare providers to meet population health needs. However, there is a substantial “know-do” gap between providers’ clinical knowledge and training (measured through clinical vignettes) and what providers do in practice (measured through standardized patients) [2–4], partially because of low motivation due to poor working and living conditions and shortages of career development and advancement opportunities [5]. While health systems try to address these issues through financial incentives and policy reforms such as performance-based financing, there is mixed evidence regarding their effectiveness [6] and substantial concerns regarding their financial sustainability in already over-stretched health systems.

As both complements and substitutes, non-financial incentives are frequently used to motivate healthcare providers to improve performance [5,7–11], drawing on providers’ mission-driven intrinsic pro-social motivation [12–15]. Pro-social motivation refers to underlying reasons why providers want to serve patients outside of financial compensation, such as their professional conscience, ethos, or dedication to serving the public’s health needs. Non-financial incentives can appeal to pro-social motivation by providing social awards and recognition or open opportunities for additional training and career advancement [12,14,16]. Their low cost is particularly attractive to budgetarily constrained health systems, but their effectiveness varies substantially across domains and settings.

Further, awards and social recognition for healthcare providers are often provided publicly, where social image considerations interact with intrinsic pro-social motivation, though whether public provision amplifies or dampens the effect of non-financial incentives is theoretically ambiguous [17]. While appealing to providers’ concern for their social image may generate additional motivation among providers who value social recognition from their peers/society, it can theoretically backfire among those discouraged by the process of “competing” for recognition or those who feel unfairly excluded or unrecognized during the awards and recognition process. Further, it is unclear whether non-financial incentives only work among those who are highly pro-socially motivated in the first place via a selection effect or whether they are effective among a broader set of providers [18].

To fill this gap, we developed and evaluated the effectiveness of a non-financial incentive using a cluster-randomized controlled trial in Tanzania among drug shopkeepers enrolled in a study evaluating the effectiveness of an intervention designed to increase the “girl-friendliness” of drug shops for sexual and reproductive health (SRH) products [19,20]. In this setting, shopkeepers are often the for-profit owner of the business and the healthcare provider with a limited set of training for dispensing over-the-counter drugs among other non-regulated health products [21]. Shopkeepers often claim to be pro-socially motivated as frontline health workers and support poor, vulnerable customers, like young women (15–24 years), by offering discounts or credit for those who are cash-constrained [22,23]. We selected this population of drug shopkeepers in Tanzania given their involvement in the parent study and the generalizability of their experience to providers in low-and middle-income countries given the prevalence of informal providers in the private sector with substantial heterogeneity in provider quality and effort. We developed a non-financial incentive that involved providing shopkeepers with monthly reports of aggregated and anonymized customer feedback from young women customers which were designed to appeal to their pro-social motivation. We randomized whether the reports were provided privately (i.e., only to the shopkeeper) or publicly as a certificate of their ratings displayed in the shop, and measured provider performance using multiple measures over 12 months. This design is similar to a non-financial incentive intervention among informal providers (hairdressers distributing female condoms) in Zambia, which found substantially improved performance (double) among those receiving publicly displayed “star thermometers” and recognition for high sales relative to those receiving financial incentives or a control arm [9]. We hypothesized that our non-financial incentive intervention would increase provider performance relative to those who do not receive the intervention, but were agnostic ex ante regarding the relative effectiveness of private versus public provision of the incentive. While the private provision of incentive is hypothesized to influence provider behavior by appealing to their pro-social motivation, public provision of the incentive additionally introduces social image concerns that may interact with pro-social motivation.

## Materials and methods

### Study design

Our cluster-randomized trial evaluated the effectiveness of a non-financial incentive in boosting health provider performance among registered drug shops (Duka La Dawa Muhimu) in Lake Zone, Tanzania that were enrolled in a parent study evaluating the effectiveness of an intervention designed to increase the “girl-friendliness” of drug shops for SRH products (NCT05357144). Details regarding the parent study are provided elsewhere [24]. All drug shops participating in the parent study were recruited for this trial between May 29 and July 5, 2023; each was informed of study procedures and data collection requirements, including both retrospective and prospective review of administrative data, and all provided written informed consent.

The study received ethics approval from the National Institute for Medical Research (NIMR/HQ/R.8a/Vol. IX/3837) in Tanzania and from the Human Research Protection Program Institutional Review Board of the University of California, San Francisco (IRB#: 21–33553; Reference #: 353456) and is preregistered on ClinicalTrials.gov (NCT05525533).

### Intervention

In this context, private drug shops are owned and often staffed by community members and serve as informal health providers that sell basic health products (e.g., over-the-counter medicines, hygiene) and provide informal consultations and diagnoses [25–27]. Drug shops provide over half of all care in underserved areas [27]. Specifically, drug shopkeepers often claim to be pro-socially motivated as frontline health workers and support poor, vulnerable customers, like young women, by offering discounts or credit [22,23].

Encouraged by the presence of pro-social motivation among shopkeepers and the potential for non-financial incentives to improve their performance, we developed a set of non-financial incentives designed to stimulate shopkeepers’ sales and distribution of SRH products to young women, targeting shopkeepers’ sense of responsibility as frontline health providers and desire to support young women identified from previous studies and in-depth qualitative interviews with shopkeepers [22,23]. Specifically, we aimed to stimulate shopkeepers’ pro-social motivation by making this aspect of their job more salient and “tug at their heart strings” using feedback from their young women customers, similar to “thank you” messages and related interventions common in the literature [5,7–11].

**Fig 1:**
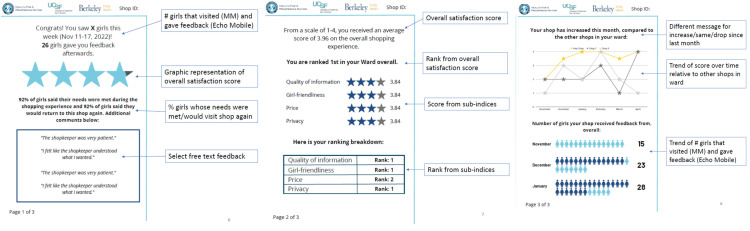
Example of customer feedback summary report.

After extensive designing, piloting and refining of the intervention with drug shopkeepers enrolled in previous studies, we converged on providing monthly summary reports of feedback from young women customers, anonymously collected through post-purchase “quick code”-based surveys. Eight questions included ratings on specific dimensions of quality (e.g., girl-friendliness, price, etc., see S1 Table for the complete “quick-code”-based survey) and a free text input to allow for appreciative “thank you” messages. We did not use validated tools from the literature to measure patient satisfaction – instead, we designed the “quick-code” based customer feedback surveys to balance what customers would be willing fill out quickly post-purchase with what would be meaningful to the drug shopkeepers. Summary reports were designed with behavioral science-informed techniques to stimulate performance by appealing to pro-social motivations for helping young women and giving areas for improvement. Fig 1 shows an example of the summary reports provided, highlighting specific design features (e.g., purposively selected encouraging free text feedback, congratulatory language highlighting the volume of young women customers providing feedback) intended to stimulate pro-social motivation. Prior to implementation and during each monthly visit when summary reports of feedback were presented to providers, the study team explained each element of the feedback report in detail and answered any questions providers had regarding the report (e.g., clarifying that the star ratings were computed based on the overall feedback score, which was the mean response to the survey question *“How satisfied were you with the overall shopping experience?”*). Providers were also trained on the questions included in the “quick-code” based surveys for full transparency and understood how the feedback scores were constructed (i.e., averages from responses from the previous month).

**Fig 2:**
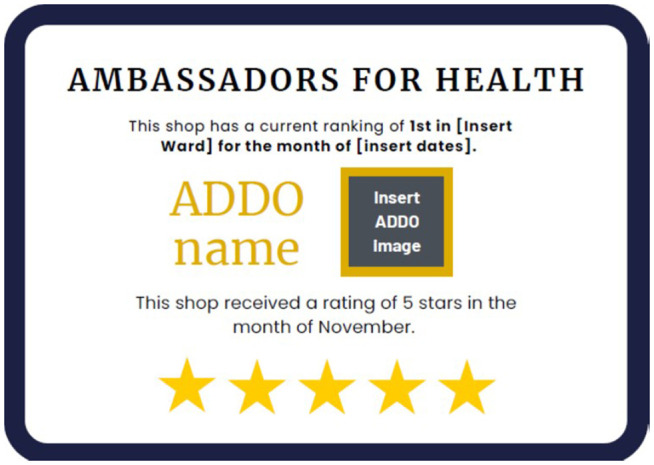
Template of publicly displayed certificate.

Recognizing the potential interaction between pro-social motivation and social image considerations in the design of non-financial incentives [16,17], we emphasized social image concerns among a subset of shops by publicly displaying results from the summary feedback reports in the shop, similar to previous examples in the literature [9]. Specifically, we designed a certificate reflecting the aggregate customer feedback score (the mean response to the survey question *“How satisfied were you with the overall shopping experience?”*) that was affixed to a wall in a shop where it would be readily visible to all (Fig 2), and updated the certificate monthly during routine field team visits. The shop’s name and shopkeeper’s photo are prominently displayed on the certificate to allow shopkeepers to reap the benefits of being publicly recognized as a young women-friendly drug shop. Further, the shop’s rank (by mean customer feedback rating that month) relative to other shops in the same geographical cluster (ward) is highlighted to stimulate competition between shops in the local area.

### Randomization

All shops enrolled in the study were previously randomized to either an Intervention or Control arm at the ward level as part of the parent study [24]. Using a phased-in design across a planned 18-month study period, we cross-randomized against the parent study intervention arms whether summary reports were provided (i) *Privately* (i.e., only presented to the shopkeeper) or (ii) *Publicl*y vis-à-vis certificates displayed in the shop in addition to summary reports given privately to the shopkeeper) to compare whether non-financial incentives are more effective when pro-social motivation is augmented by social image considerations.

Table 1 shows the original design for the study: 41 wards from the parent study were randomized to Groups 1, 2 and 3 respectively, stratifying by geographical region (2 regions) and parent study intervention arm (2 arms) only. We did not implement any allocation concealment. To maximize balance along observable characteristics at baseline, we conducted a re-randomization procedure where we randomly assigned wards across the three groups 5,000 times and picked the most balanced treatment assignment (maximizes the minimum p-value in the balance tests, S2 Table). There were no chance imbalances on any observable characteristics (p = 0.9763 from omnibus test of joint orthogonality).

**Table 1:** Timeline for group assignment.

Group Assignment	Phase 0: Pre-intervention (July 2022 – June 2023)	Phase 1: Initial condition (June – December 2023)	Phase 2: Secondary condition^1^
	***0-6 months***	***6-12 months***	***12-18 months***
Group 1 (N = 12 wards)	No feedback	No feedback	Private feedback
Group 2 (N = 15 wards)	No feedback	Private feedback	Public feedback
Group 3 (N = 14 wards)	No feedback	Public feedback	Public feedback

^1^Due to unanticipated changes in the government oversight structure in Tanzania, the parent study was terminated prematurely in January 2024, approximately 6 months ahead of schedule, removing Phase 2 of our original research design. We also did not implement a planned awards/recognition ceremony under the Public feedback arm as a result.

For an initial six months (Phase 0; July 2022 – June 2023), all shops received *No feedback* while shop-level outcomes data on performance were collected. Rollout by ward was staggered across geographical regions. Afterwards (Phase 1; June – December 2023), shops were randomly assigned to receive *No feedback, Private feedback,* and *Public feedback* respectively for six months. Originally, we had planned to change shops’ intervention exposure assignment after another six months (Phase 2) to generate sufficient experimental variation to estimate the effect of Private vs Public feedback separately. However, due to unanticipated changes in the government oversight structure in Tanzania, the parent study was terminated prematurely in January 2024, approximately 6 months ahead of schedule, removing Phase 2 of our original research design.

### Data sources

At baseline of the parent study, all study participants were administered structured surveys that included modules on shop and shopkeeper characteristics, business practices, sales, prices and revenues, and shopkeeper pro-social motivation and social image concerns. We measured pro-social motivation (altruism, positive reciprocity and trust) through experimentally validated questions from the Global Preferences Survey [28,29], an experimentally validated survey module against common lab-in-the-field experiments and tested across 80,000 people in 76 countries. To measure a) altruism, we asked participants i) to rate their willingness to give to good causes from a scale of 0–10; if they unexpectedly received 100,000 TZS, how much they would be willing to donate to ii) an unspecified good cause, iii) an organization that supported SRH, and iv) an organization that supported HIV prevention, respectively. To measure b) positive reciprocity, we asked participants i) to rate their willingness to return a favor from a scale of 0–10; ii) how much they would be willing to give as a “thank you” gift to someone who did them favor. To measure c) trust, we asked participants to rate to what extent they assumed that people have only the best intentions from a scale of 0–10. All questions involving hypothetical monetary stakes used amounts from the original survey module, inflated to reflect prices at the time of data collection. We measured respondents’ concern for their social image using a series of questions eliciting shopkeepers’ beliefs regarding the importance of being well regarded by fellow shopkeepers, being seen as a socially responsible shopkeeper, and being seen as a supporter of young women respectively (S3 Table).

As the basis for the summary reports provided to shopkeepers as a non-financial incentive, we collected feedback from young women customers at each shop. While this data is likely collected from a select sample (young women customers could opt into filling out the “quick-code” survey and shopkeepers could potentially influence which young women customers to respond), the number of responses is nonetheless informative regarding shopkeepers’ engagement with the non-financial incentive.

As part of their enrollment in the parent study, all drug shops kept records of individual transactions using a digital point-of-sale tracking and inventory management system operated on tablet devices given to each shop. Specifically, for every transaction occurring at the drug shop, shopkeepers recorded the item(s) sold, the price it was sold for, and whether it was sold to an young women customer. Shopkeepers underwent extensive training at the beginning of the parent study to ensure accurate data entry and were briefed regarding the business-related benefits of maintaining accurate administrative records. Restocking and reimbursement for products distributed to young women in the parent trial were contingent on accurate record-keeping, which was routinely monitored and randomly audited as a stipulation of study participation.

### Outcomes

The primary outcomes were the quantities of i) all products, ii) HIV self-test kits, and iii) other sexual and reproductive health products sold to young women customers per shop per month. These outcomes reflect measures of aggregate provider performance in our study context. Secondary outcomes were the quantities of specific sexual and reproductive health products, including i) condoms, ii) emergency contraception, iii) oral contraception, and iv) pregnancy tests sold to young women customers per shop per month, reflecting disaggregated measures of provider performance.

### Statistical analysis

We designed the study to detect a 20% relative increase for the primary outcomes of HIV self-test kits and contraceptives sold to young women customers aggregated in biweekly increments with 80% power, assuming α = 0.05, ICC = 0.23 and N= = 4,320 shop-time observations based on 36 biweekly periods at 120 shops over an 18-month study period. However, our statistical power was reduced for two reasons. First, the early termination of the parent study at 12 months negated the planned Phase 2 exposures. Second, sales volumes per shop were lower than anticipated, requiring us to aggregate shop-specific sales data at the monthly level instead to yield meaningful continuous variation.

First, we estimated descriptive statistics on shopkeeper characteristics and our primary and secondary outcomes. We tested for differences in means on shopkeeper characteristics at baseline of the parent study by Group (pairwise comparisons between those receiving *No Feedback*, *Private*, and *Public* in Phase 2). We descriptively graphed how outcomes varied by Group across the entire study period and by study month. Second, using an intention-to-treat approach, we estimated linear regression models between Group (receiving *No Feedback*, *Private* and *Public* in Phase 2) and each primary and secondary outcome, respectively, controlling for the parent study treatment arm and time trends by including linear, quadratic and cubic terms given expected seasonality in our outcomes. We clustered standard errors at the shop level. As robustness checks, we i) estimated models transforming the outcome with inverse hyperbolic sine to address right-skewness [30], ii) controlled for shop level fixed effects, and iii) estimated models on the extensive margin (by transforming each outcome into a binary indicator for any sales during that shop-month). Third, we examined how shopkeeper performance varied by pro-social motivation and concern for social image. Specifically, we plotted how mean sales per shop per month varied by quintiles of z-scored measures for pro-social motivation and concern for social image. Finally, we plotted how mean sales per shop per month varied by quintiles of customer feedback ratings and volume of surveys received. All analyses were done using Stata V17.0.

### Inclusivity in global research

Additional information regarding the ethical, cultural, and scientific considerations specific to inclusivity in global research is included in the Supporting information.

## Results

**Fig 3:**
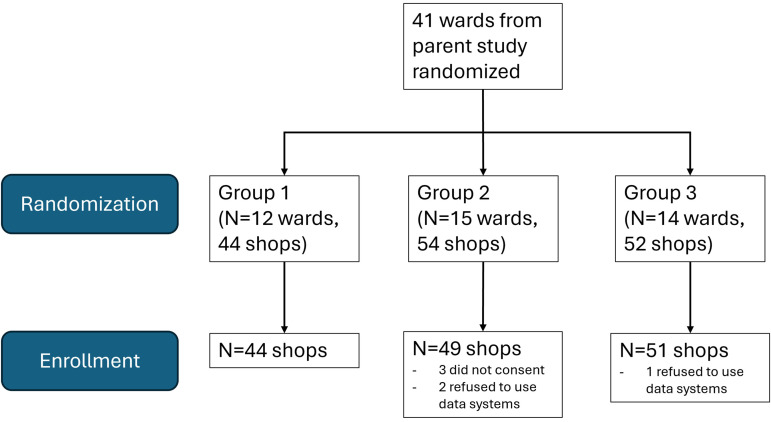
Consort diagram.

150 drug shops across 41 wards from the parent study were recruited (Fig 3). 144 (96%) were enrolled and included in the analysis: 3 shops did not consent, and 2 shops refused to use data collection systems required for the study.

Table 2 shows descriptive statistics on sales per shop per month to young women customers. Between July 2022 and December 2023, 1,055,059 products were sold across 144 participating shops, contributing 2,136 shop-month observations for analysis. Shops sold a mean (median) of 194 (106) products per month, including a mean of 10 HIV self-test kits and 17 sexual and reproductive health products. Shops made a mean of 62 USD in revenue per month from young women customers.

**Table 2:** Descriptive characteristics on shopkeeper characteristics and sales.

Sales per shop per month to young women customers	Mean	Median	SD	Min	Max	N
Products sold	193.66	106	240.99	0	2279	2136
HIV self-test kits	9.90	7	9.50	0	65	2136
Sexual and Reproductive Health Products	16.61	2	41.72	0	541	2136
• Condoms	4.81	0	17.64	0	274	2136
• Emergency contraception	2.53	0	7.64	0	123	2136
• Oral contraception	4.90	0	15.06	0	138	2136
• Pregnancy tests	4.36	0	9.58	0	132	2136
Revenue (USD) from young women sales	62	22	120	0	1560	2136
**Pro-Social Motivation and Concern for Social Image**	**Mean**	**Median**	**SD**	**Min**	**Max**	**N**
**Positive Reciprocity**						
**•** Self-assessment: willingness to return a favor (0–10)^#^	9.10	10.00	1.34	4.00	10.00	141
• Gift in exchange for help (0–15,000 TZS)	0.94	1.00	0.25	0.00	1.00	141
• As proportion of cost	100.19	100.00	39.65	0.00	150.00	131
**Altruism**						
**•** Self-assessment: willingness to give to good causes (0–10) ^#^	8.37	9.00	1.91	0.00	10.00	141
• Donation decision (% of total endowment)	22.80	20.00	18.32	0.00	100.00	141
• To an organization that supports SRH (% of total endowment)	17.91	10.00	18.36	0.00	100.00	141
• To an organization that supports HIV prevention (% of total endowment)	14.98	10.00	16.55	0.00	100.00	141
**Trust**						
**•** Assume best intentions (0–10) ^#^	6.49	7.00	3.37	0.00	10.00	141
**Social Image**						
It is important to me that I am well regarded by fellow drug shopkeepers	7.71	8.00	2.37	1.00	10.00	141
It is important to me that other drug shopkeepers see me as socially responsible shopkeeper	7.62	8.00	2.47	0.00	10.00	141
It is important to me that other drug shopkeepers think that I support young women	7.82	8.00	2.37	0.00	10.00	141
**Customer Feedback**	**Mean**	**Median**	**SD**	**Min**	**Max**	**N**
Volume of customer feedback per shop	92	38	282	1	2726	99
Mean feedback score (1–4) per shop ^##^	3.51	3.55	0.34	2.17	4	99

^#^ All self-assessment questions were scaled from 0–10, with 10 as the highest value for each pro-social motivation measure and 0 as the lowest.

^##^ This refers to the mean response to the survey question *“How satisfied were you with the overall shopping experience?”*

Table 2 also shows descriptive statistics on shopkeeper pro-social motivation and concern for their social image. From self-assessment questions (scaled from 0–10, 10 as the highest value for each pro-social motivation measure and 0 as the lowest), shopkeepers demonstrated high levels of positive reciprocity (9.10/10), altruism (8.37/10), and moderate levels of trust (6.49). Shopkeepers were willing to give a thank-you gift 94% of the time, worth 100% of the cost of help received (positive reciprocity), and donate 23% of an unexpected monetary gain to a good cause (altruism). The share of unexpected monetary gain donated were 18% and 15% when the cause was specified as supporting sexual and reproductive health and HIV respectively. Shopkeepers also showed moderate levels of concern for their social image. Shopkeeper pro-social motivation and concern for their social image did not differ by Group (S2 Table). Young women customers completed 9,108 anonymous surveys across 99 shops. Shops received a mean (median) of 92 (38) surveys over the study period and a mean overall score of 3.5 from a Likert scale of 1–4 (4 as the highest value, 1 as the lowest).

**Fig 4:**
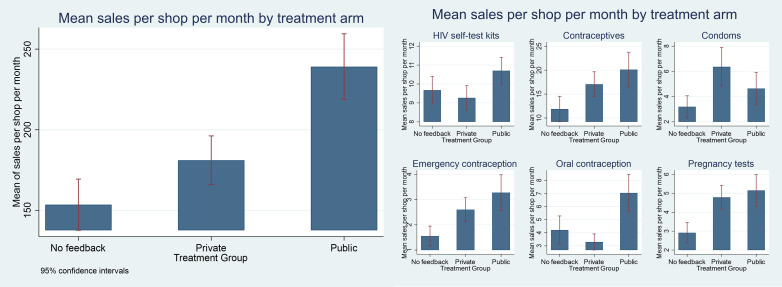
Mean sales per shop per month by treatment arm.

Fig 4 shows how each outcome varied by treatment arm across the study period. Compared to shops that received no feedback (153, 95% CI: 137, 169), mean sales per shop per month were higher among shops that received private (181, 95% CI: 166, 196) and public feedback (239, 95% CI: 219, 259) respectively. Differences in shopkeeper performance by treatment arm were larger for sexual and reproductive health products compared to HIV self-test kits. These differences persisted over time during the study period (S4 Fig).

Table 3 shows results from regression models on the impact of *Private* and *Public* feedback on our primary outcomes, relative to shops that received *No Feedback*. *Private feedback* did not increase provider performance across various measures. However, *Public* feedback increased sales by 113 products per shop per month (95% CI: 38, 189), a 58% increase relative to a mean of 194 products across the study population. This was most notable for sexual and reproductive health products (96%, 95% CI: 4, 187), specifically oral contraception (154%, 95% CI: 9, 306) and pregnancy tests (75%, 95% CI: 8%, 143%) (S5 Table). These results are qualitatively similar under robustness checks transforming the outcome with inverse hyperbolic sine (S6 Table). Only the result on sales overall holds when exclusively examining extensive margin effects (S7 Table). The results are qualitatively similar but no longer statistically significant with the inclusion of shop level fixed effects due to the substantially reduced statistical power (S8 Table).

**Table 3:** Impact of customer feedback on overall HIVST/contraceptives distributed.

N = 2136	Performance Measure		
	Quantities of all products sold	HIV self-test kit sold	SRH products sold
**Group**			
• No feedback (ref)	–	–	–
• Private	47 [24%] (−18, 113)	0.17 [2%] (−1.87, 2.24)	7.58 [46%] (−2.92, 18)
• Public	113** [58%] (38, 189)	1.55 [16%] (−0.73, 3.82)	16* [96%] (0.64, 31)
Outcome mean	194	9.90	16.61
Intraclass correlation coefficient (ICC)	0.350	0.160	0.339
R2	0.06	0.06	0.14

*p < 0.05, **p < 0.01, **p < 0.001. Coefficients and 95% confidence intervals in brackets. Effect sizes as a percentage of the outcome mean presented in square brackets. All models control for parent study treatment arm and time trends by including linear, quadratic and cubic terms accordingly.

**Fig 5:**
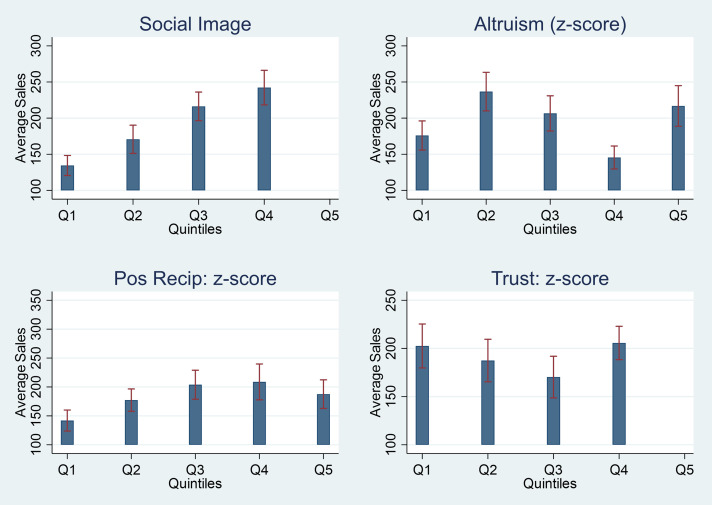
Heterogeneity by pro-social motivation and concern for social image.

Fig 5 shows mean sales per shop per month by quintiles of pro-social motivation and concern for social image. While mean sales did not substantially vary by levels of altruism, positive reciprocity or trust, mean sales were positively correlated with shopkeepers’ concern for their social image across multiple measures. Examining different measures of social image separately show similar patterns regarding the importance of being well regarded by fellow shopkeepers, being seen as a socially responsible shopkeeper, and being seen as a supporter of young women (S9 Fig).

**Fig 6:**
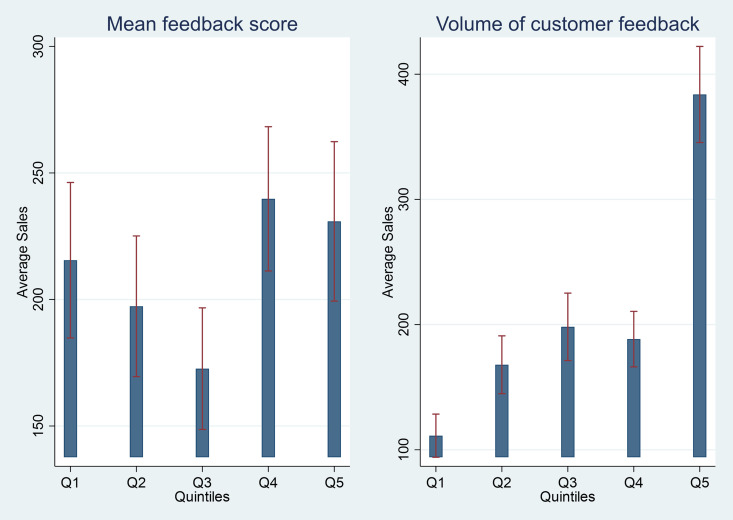
Heterogeneity by quality and volume of customer feedback received.

Fig 6 shows mean sales per shop per month by quintiles of customer feedback ratings (mean feedback score) and volume (number of “quick code” surveys received) of customer feedback received. While mean sales do not substantially vary by customer feedback ratings, mean sales are positively correlated with the volume of customer feedback received.

## Discussion

In our cluster-randomized trial among drug shopkeepers in Tanzania, we found suggestive evidence regarding the effectiveness of a non-financial incentive in boosting provider performance. Specifically, we found that providing summary reports of customer feedback from young women increased shopkeepers’ sales to young women customers. We contribute to the literature on approaches to motivate providers in low-and middle-income countries [10] by adding to the evidence base for non-financial incentives [7–9,11,13], a promising approach even taking into consideration the mixed evidence given the low cost and ease of implementation among resource-constrained health systems.

We provide some of the first evidence exploring the mechanisms behind non-financial incentives using an innovative study design combined with survey data measuring pro-social motivation and providers’ concern for their social image. Non-financial incentives such as awards and social recognition are often provided publicly, where social image considerations interact with intrinsic pro-social motivation, though whether this amplifies or dampens the effect of non-financial incentives is theoretically ambiguous [17]. While appealing to providers’ concern for their social image may generate additional motivation among providers who value social recognition from their peers/society, it can theoretically backfire among those discouraged by the process of “competing” for recognition or those that feel unfairly excluded or unrecognized during the awards/recognition process. We tested this empirically by varying whether non-financial incentives were provided privately or public in our context and found that public incentives were overwhelmingly more effective at boosting performance, similar to the result among hairdressers in Zambia (9). This allays theoretical concerns around public incentives backfiring, or at least suggests that additional motivation leveraging social image concerns dominate any negative effects among those discouraged by the process. While we attribute the effectiveness of public incentives to the interaction of social image concerns with pro-social motivation based on our ex-ante hypothesis, we cannot rule out the existence of other potential mechanisms explaining this result including an increase in public accountability given the publicly displayed certificate.

Further, we found that shopkeepers’ concern for their social image at Baseline was positively correlated with performance while pro-social motivation (altruism, positive reciprocity or trust) was not, even though the non-financial incentive was designed specifically to appeal to providers’ pro-social motivation. Combined with the effectiveness of the public arm relative to the private arm, this suggests that the non-financial incentive primarily boosted provider performance by stimulating social comparisons in performance between providers, especially among those who were more concerned about their social image to begin with. This is also consistent with the finding that mean sales were positively correlated with volume of customer feedback received – as providers may convince more young women customers into providing feedback to improve their reputation – but not correlated with the mean feedback scores – which are harder to manipulate since they are submitted confidentially by young women customers via “quick-code”. This has important implications for the design of non-financial incentives in the future, suggesting that purposefully leveraging provider’s concern for their social image in could amplify the effectiveness of non-financial incentives in boosting provider performance.

Finally, we also contribute to the growing literature on provider motivation in low and middle-income countries. Using similar measures of altruism, researchers in Kenya found that more altruistic providers were less likely to report false-positive malaria tests and sell unnecessary malaria drugs [31,32] and less likely to promote injectable contraceptives when faced with potentially competing financial incentives. In India, researchers found that providers under-prescribe oral rehydration salts for diarrhea in favor of antibiotics not exclusively because of perverse financial incentives, but because they misperceive that patients do not want oral rehydration salts [4]. Our study adds to this evidence base on providers being motivated by non-financial incentives, demonstrating the feasibility of interventions designed to boost provider performance by leveraging these factors. Given their low cost and ease of maintenance via automated systems once initial set-up and implementation is completed, it is feasible and sustainable to scale up similar interventions within national health systems. However, further research is needed to understand whether treatment effects are sustained over time and how the incentive design needs to be tailored over time to reduce habituation among providers.

Our study has several limitations. First, our study is limited in statistical power due to the early termination of the parent study, which also prevented us from testing whether the effectiveness of non-financial incentives fade over time or whether *Public* increases performance among providers previously exposed to *Private*. Correcting for multiple hypothesis testing using Benjamini-Hochberg p-values attenuates several marginally significant findings (S10 Table). Nonetheless, the effect size from *Public* was meaningful (58% increase in sales relative to *No feedback*) despite the wide confidence intervals, especially given the low cost of the intervention. Further, we did not see a drop off in the relative differences between treatment arms within our reduced study period, suggesting the possibility of longer-term persistence. However, the follow-up period within each phase (6-months) remains short which limits our ability to examine longer-term effects on provider behavior change. Second, the customer feedback survey data does not come from a representative sample of young women customers and we cannot assess selection into responding, which particularly affects the interpretation of results from our heterogeneity analysis. However, while providers could potentially manipulate customer feedback scores through fraud, we do not see evidence of that when examining metadata on the “quick-code” surveys (e.g., suspiciously many surveys submitted from the same phone numbers) and believe that providers taking the time and effort to manipulate the reports would be an indication that the non-financial incentives were valuable to the providers in the first place. Further, we do not see differences in mean survey response rates by treatment arm (12% in both Private and Public, S11 Table) suggesting that selection bias does not differ by treatment arm. Third, our analysis on the relationship between provider performance and baseline pro-social motivation and concern for social image is correlational, since we are not powered to detect treatment heterogeneity by pro-social motivation/concern for social image. Nonetheless, this correlational evidence is consistent with the main result regarding the effectiveness of *Public* and the sheds light on the potential mechanisms behind its effectiveness. Fourth, given the limitations of our data, we are only able to measure average treatment effects on provider performance and cannot rule out the possibility of unintended consequences such as demotivation among lower performers and manipulation of performance indicators to generate more favorable summary feedback reports. However, given that the intervention emphasizes the overall feedback score from customer feedback surveys by design rather than performance measures such as our primary outcomes in both the summary report and publicly displayed certificates, there is limited incentive for providers to manipulate their performance metrics via administrative data on individual transactions collected via their point-of-sale tracking and inventory management system. Fifth, our study is potentially affected by Hawthorne effects due to the high visibility of the non-financial incentive and the regular interaction between the study team and providers to present the summary feedback report/publicly displayed certificate. Treatment effects may be reduced when the intervention is implemented at scale with fewer regular touch points with providers. Finally, even though informal providers in the private sector facing similar incentives are common across low-and middle-income countries, further research is needed to confirm our study’s external validity beyond the Tanzanian context. Further research is needed to understand the nature of pro-social motivation in other contexts to adapt the intervention accordingly to maximize effectiveness.

## Conclusion

Publicly provided, low-cost non-financial incentives increased performance among drug shopkeepers in Tanzania serving young women. In addition, provider performance was correlated with provider’s concern for their social image at baseline, rather than pro-social motivation. Future interventions using non-financial incentives to motivate healthcare providers should consider leveraging providers’ concern for their social image to amplify the effectiveness of incentives. Effective implementation of similar interventions can complement broader structural health reforms to maximize output from scarce providers and improve population health outcomes. Further research is needed to understand whether treatment effects are sustained over time and whether treatment sequencing affects performance outcomes given the limitations of our study.

## Supporting information

S1 Table USSD-based customer feedback survey. (PDF)

S2 Table Balance of baseline drug shop characteristics by study arm. (PDF)

S3 Table Survey instrument for pro-social motivation and social image. (PDF)

S4 Fig Mean sales per shop per month by treatment arm over time. (PDF)

S5 Table Impact of customer feedback on secondary outcomes. (PDF)

S6 Table Robustness check with inverse hyperbolic sine. (PDF)

S7 Table Robustness check with extensive margin. (PDF)

S8 Table Robustness check with shop level fixed effects. (PDF)

S9 Fig Heterogeneity by social image dimensions. (PDF)

S10 Table Impact of customer feedback on overall HIVST/contraceptives distributed with multiple hypothesis testing corrections. (PDF)

S11 Table Comparison of customer feedback survey response rates by treatment arm. (PDF)

S1 File ProSociality Protocol. (PDF)

S2 File Inclusivity in global research questionnaire. (PDF)
