# Supplementary material for: Boosting health provider performance with non-financial incentives: A cluster-randomized controlled trial in Tanzania
Source: PLoS One. 2025 Sep 11;20(9):e0330989. doi: 10.1371/journal.pone.0330989 (PMC12425186; doi:10.1371/journal.pone.0330989)
Supplement: S1 Table — (PDF) [file pone.0330989.s001.pdf]

Table S1: USSD-based customer feedback survey

Customer Feedback Survey (*149*46*20#)

1. How satisfied were you with the **overall shopping experience**?
2. How satisfied were you with the **quality of information** provide by the shop?
3. How satisfied were you with the **girl-friendliness** of the shop?
4. How satisfied were you with the **prices** of the products you purchased?
5. How satisfied were you with the **privacy** of your interaction with the shopkeeper?
6. Were all of your needs met during shopping experience? (Yes/No)
7. Would you return to this shop again? (Yes/No/Maybe)
8. Please leave any comments or suggestions (free text)
