# Supplementary material for: Boosting health provider performance with non-financial incentives: A cluster-randomized controlled trial in Tanzania
Source: PLoS One. 2025 Sep 11;20(9):e0330989. doi: 10.1371/journal.pone.0330989 (PMC12425186; doi:10.1371/journal.pone.0330989)
Supplement: S2 Table — (PDF) [file pone.0330989.s002.pdf]

Table S2: Balance of baseline drug shop characteristics by study arm

Variable	Mean			Diff						Joint orthogonality
	Group 1	Group 2	Group 3	2 vs 1	p-value	3 vs 1	p-value	3 vs 2	p-value	p-value
Number of shops	3.308	3.333	3.308	0.03	0.927	1.000	0.913	-0.03	0.927	0.994
Mean sales per shop	3955.756	4205.378	3371.647	249.62	0.785	0.557	0.109	-833.73	0.377	0.661
Mean young women sales per shop	1675.103	1495.889	1343.974	-179.21	0.699	0.578	0.515	-151.92	0.723	0.805
Mean share of young women sales per shop	0.425	0.459	0.401	0.03	0.585	0.710	0.758	-0.06	0.412	0.664
Mean HIVST distributed per shop	88.109	90.172	88.179	2.06	0.877	0.997	0.586	-1.99	0.900	0.987
Mean SRH product distributed per shop	145.840	157.094	137.808	11.25	0.887	0.917	0.100	-19.29	0.794	0.967
Mean condoms distributed per shop	51.827	55.761	45.353	3.93	0.891	0.820	0.045*	-10.41	0.670	0.926
Mean EC distributed per shop	19.212	18.506	16.429	-0.71	0.963	0.852	0.805	-2.08	0.880	0.981
Mean OCP distributed per shop	33.135	36.372	29.641	3.24	0.898	0.881	0.027	-6.73	0.761	0.959
Mean UPT distributed per shop	41.667	46.456	46.385	4.79	0.791	0.800	0.504	-0.07	0.997	0.958
Mean revenue per shop	1813.270	1995.422	1730.022	182.15	0.689	0.871	0.441	-265.40	0.640	0.863

*p<0.05; **p<0.01; ***p<0.001. P-values from regressions of each variable on treatment assignment controlling for strata fixed effects. P=0.9763 from omnibus test of joint orthogonality.

Variable	Mean			Diff					
	Group 1	Group 2	Group 3	2 vs 1	p-value	3 vs 1	p-value	3 vs 2	p-value
**Positive Reciprocity**									
• Self-assessment: willingness to return a favor (0-10)	9.09	9.04	9.16	0.05	0.872	-0.07	0.815	-0.11	0.670
• Gift in exchange for help (0-15,000 TZS)	0.98	0.89	0.94	0.09	0.100	0.04	0.373	-0.05	0.385
• As proportion of cost	110.47	89.38	100.00	21	0.015*	10.47	0.186	-10.63	0.230
**Altruism**									
• Self-assessment: willingness to give to good causes (0-1)	8.70	8.20	8.24	0.51	0.187	0.47	0.218	-0.04	0.923
• Donation decision (% of total endowment)	26.25	20.87	21.57	5.38	0.176	4.68	0.225	-0.70	0.847

• To an organisation that supports SRH (% of total endowment)	21.59	14.91	17.45	6.68	0.091	4.14	0.281	-2.54	0.487
• To an organisation that supports HIV prevention (% of total endowment)	14.20	16.57	14.22	-2.36	0.544	-0.01	0.997	2.35	0.515
**Trust**									
• Assume best intentions (0-10)	6.50	6.67	6.31	-0.17	0.813	0.19	0.798	0.36	0.584
**Social Image**									
It is important to me that I am well regarded by fellow drug shopkeepers	7.41	7.37	8.27	0.04	0.941	-0.87	0.066	-0.90	0.057
It is important to me that other drug shopkeepers see me as socially responsible shopkeeper	7.45	7.04	8.27	0.41	0.473	-0.82	0.083	-1.23	0.014*
It is important to me that other drug shopkeepers think that I support young women	7.89	7.24	8.29	0.65	0.252	-0.41	0.349	-1.06	0.030*

*p<0.05; **p<0.01; ***p<0.001. P-values from regressions of each variable on Group assignment. P=0.591 from omnibus test of joint orthogonality.
