# Supplementary material for: Boosting health provider performance with non-financial incentives: A cluster-randomized controlled trial in Tanzania
Source: PLoS One. 2025 Sep 11;20(9):e0330989. doi: 10.1371/journal.pone.0330989 (PMC12425186; doi:10.1371/journal.pone.0330989)
Supplement: S3 Table — (PDF) [file pone.0330989.s003.pdf]

Table S3: Survey instrument for pro-social motivation and social image

Q85 We now ask you for your willingness to act in a certain way. Please again indicate your answer on a scale from 0 to 10. A 0 means “completely unwilling to do so,” and a 10 means “very willing to do so.” You can also use any number between 0 and 10 to indicate where you fall on the scale, using 0, 1, 2, 3, 4, 5, 6, 7, 8, 9, or 10.

Q88 How willing are you to give to good causes without expecting anything in return? (Altruism WP13421R) _________________(0-10)

Q89 How well do the following statements describe you as a person? *Hint: Please indicate your answer on a scale from 0 to 10. A 0 means “does not describe me at all” and a 10 means “describes me perfectly”. You can also use any numbers between 0 and 10 to indicate where you fall on the scale, like 0, 1, 2, 3, 4, 5, 6, 7, 8, 9, 10*

Q90.When someone does me a favor, I am willing to return it (Positive Reciprocity WP13422R)

_________________(0-10)

Q91 I assume that people have only the best intentions (Trust WP13424
_________________(0-10)

Q92 It is important to me that I am well regarded by my fellow drug shopkeepers

_________________(0-10)

Q93. It is important to me that other drug shopkeepers see me as a socially responsible shopkeeper _________________(0-10)

Q94. It is important to me that other drug shopkeepers think that I support adolescent girls and young women _________________(0-10)

Q95a Please think about what you would do in the following situation. You are in an area you are not familiar with, and you realise that you lost your way. You ask a stranger for directions. The stranger offers to take you to your destination. Helping you costs the stranger about **10,000 TZS** in total. However, the stranger says he or she does not want any money from you. You have 6 presents with you. The cheapest present costs **2,500 TZS**, the most expensive one costs **15,000** TZS. Do you give one of the presents to the stranger as a “thank-you” gift?

- ◯ No (1) ***Skip to 96***
- ◯ Yes (2)

Q95b Which present do you give to the stranger? (0, 2500, 5000, 7500, 10000, 12500, 15000) (Positive Reciprocity WP13458R)

- No, would not give present (1)
- The present worth **2,500 TZS** (2)
- The present worth **5,000 TZS** (3)
- The present worth **7,500 TZS** (4)
- The present worth **10,000 TZS** (5)
- The present worth **12,500 TZS** (6)
- The present worth **15,000 TZS** (7)
- Don't know / No response (99)
- Refused (98)

Q96 Imagine the following situation: today you unexpectedly receive **100,000 TZS**. How much of this amount would you donate to a good cause? (Altruism WP13459R)

Values between 0 and 100,000 are allowed

- TSH (1) ________________________

Q97 How much of this amount would you donate to an organization that supports adolescent girls and young women access sexual and reproductive health services?

- TSH (1) ________________________

Q98 How much of this amount would you donate to an organization that supports HIV prevention?

- TSH (1) ________________________
