# Supplementary material for: Boosting health provider performance with non-financial incentives: A cluster-randomized controlled trial in Tanzania
Source: PLoS One. 2025 Sep 11;20(9):e0330989. doi: 10.1371/journal.pone.0330989 (PMC12425186; doi:10.1371/journal.pone.0330989)
Supplement: S4 Fig — (PDF) [file pone.0330989.s004.pdf]

Figure S4: Mean sales per shop per month by treatment arm over time

a) **Quantities of all products sold**

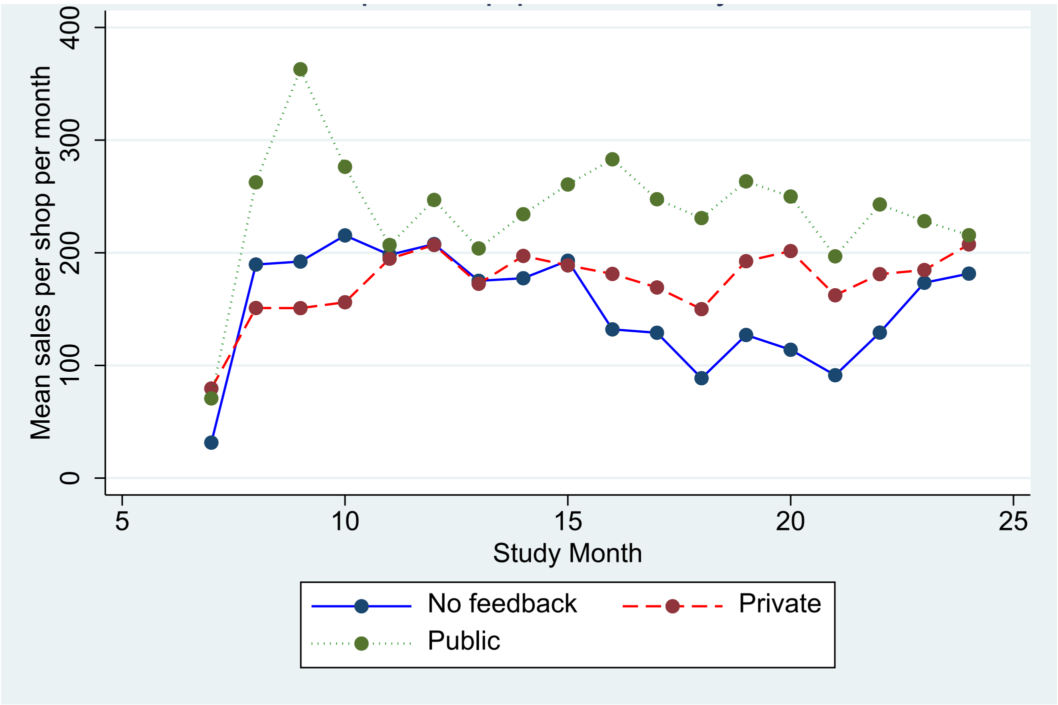

b) **HIV self-test kits sold**

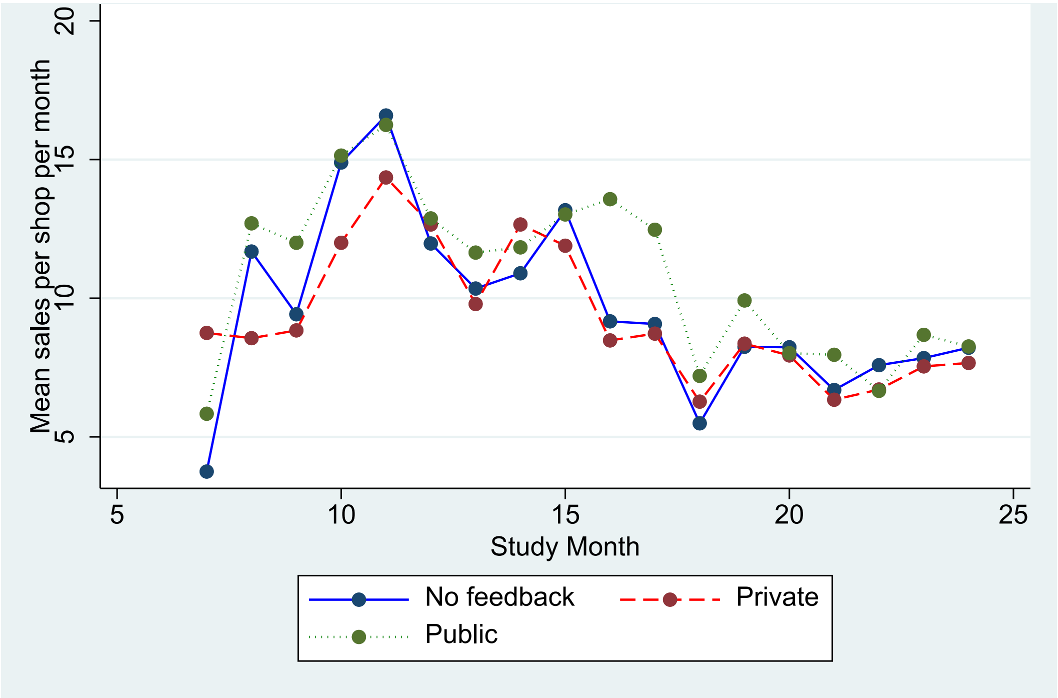

c) **SRH products sold**

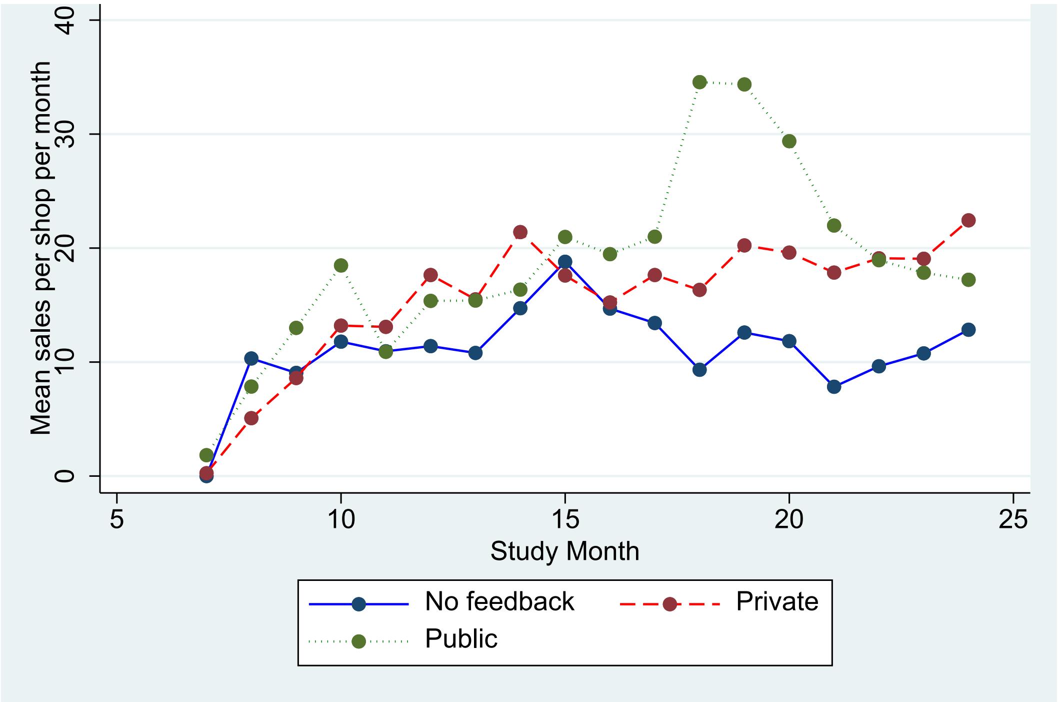
Mean sales per shop per month
0
10
20
30
40
5
10
15
20
25
Study Month
No feedback
Private
Public
