# Supplementary material for: Boosting health provider performance with non-financial incentives: A cluster-randomized controlled trial in Tanzania
Source: PLoS One. 2025 Sep 11;20(9):e0330989. doi: 10.1371/journal.pone.0330989 (PMC12425186; doi:10.1371/journal.pone.0330989)
Supplement: S5 Table — (PDF) [file pone.0330989.s005.pdf]

Table S5: Impact of customer feedback on secondary outcomes

N=2136	**Performance Measure**			
	**Condoms sold**	**Emergency Contraception sold**	**Oral contraception sold**	**Pregnancy tests sold**
**Group**				
- No feedback	-	-	-	-
- Private	3.63<br>(-1.37, 8.63)	1.32<br>(-0.36, 3.01)	0.17<br>(-3.38, 3.72)	2.46<br>(-0.11, 5.02)
- Public	1.50<br>(-1.50, 4.50)	3.38<br>(-0.54, 7.30)	7.57*<br>(0.42, 15)	3.29*<br>(0.33, 6.25)
Outcome mean	4.81	2.53	4.90	4.37
R2	0.07	0.13	0.13	0.13

*p<0.05, **p<0.01, **p<0.001. Coefficients and 95% confidence intervals in brackets.
