# Supplementary material for: Boosting health provider performance with non-financial incentives: A cluster-randomized controlled trial in Tanzania
Source: PLoS One. 2025 Sep 11;20(9):e0330989. doi: 10.1371/journal.pone.0330989 (PMC12425186; doi:10.1371/journal.pone.0330989)
Supplement: S6 Table — (PDF) [file pone.0330989.s006.pdf]

Table S6: Robustness check with inverse hyperbolic sine

| N=2136 | **Primary Outcome** | | | **Secondary Outcome** | | | |
|---|---|---|---|---|---|---|---|
| | **Quantities of all products sold** | **HIV self-test kit sold** | **SRH products sold** | **Condoms sold** | **Emergency Contraception sold** | **Oral contraception sold** | **Pregnancy tests sold** |
| **Group** | | | | | | | |
| - No feedback | - | - | - | - | - | - | - |
| - Private | 0.41<br>(-0.15, 9.96) | 0.20<br>(-0.17, 0.57) | 0.54*<br>(0.07, 1.01) | 0.43*<br>(0.06, 0.80) | 0.31<br>(-0.01, 0.64) | 0.20<br>(-0.16, 0.57) | 0.54**<br>(0.17, 0.91) |
| - Public | 0.90***<br>(0.39, 1.41) | 0.34<br>(-0.04, 0.72) | 0.52*<br>(0.00, 1.04) | 0.23<br>(-0.17, 0.63) | 0.35<br>(-0.05, 0.75) | 0.41<br>(-0.09, 0.92) | 0.54**<br>(0.17, 0.91) |
| R2 | 0.07 | 0.06 | 0.30 | 0.21 | 0.24 | 0.23 | 0.23 |

*p<0.05, **p<0.01, **p<0.001. Exponentiated coefficients and 95% confidence intervals in brackets.
