# Supplementary material for: Boosting health provider performance with non-financial incentives: A cluster-randomized controlled trial in Tanzania
Source: PLoS One. 2025 Sep 11;20(9):e0330989. doi: 10.1371/journal.pone.0330989 (PMC12425186; doi:10.1371/journal.pone.0330989)
Supplement: S7 Table — (PDF) [file pone.0330989.s007.pdf]

Table S7: Robustness check with extensive margin

N=2136	**Primary Outcome**			**Secondary Outcome**			
	**Quantities of all products sold**	**HIV self-test kit sold**	**SRH products sold**	**Condoms sold**	**Emergency Contraception sold**	**Oral contraception sold**	**Pregnancy tests sold**
**Group**							
- No feedback	-	-	-	-	-	-	-
- Private	0.03<br>(-0.04, 0.11)	0.09<br>(-0.01, 0.19)	0.08<br>(-0.04, 0.20)	0.12*<br>(0.00, 0.25)	0.11<br>(-0.00, 0.23)	0.07<br>(-0.04, 0.19)	0.15*<br>(0.02, 0.27)
- Public	0.07*<br>(0.01, 0.14)	0.08<br>(-0.03, 0.19)	0.11<br>(-0.01, 0.23)	0.04<br>(-0.08, 0.16)	0.07<br>(-0.05, 0.19)	-0.00<br>(-0.13, 0.12)	0.18**<br>(0.06, 0.31)
Outcome mean	0.95	0.84	0.60	0.36	0.25	0.28	0.49
R2	0.03	0.02	0.16	0.15	0.26	0.22	0.18

*p<0.05, **p<0.01, **p<0.001. Coefficients and 95% confidence intervals in brackets.
