# Supplementary material for: Boosting health provider performance with non-financial incentives: A cluster-randomized controlled trial in Tanzania
Source: PLoS One. 2025 Sep 11;20(9):e0330989. doi: 10.1371/journal.pone.0330989 (PMC12425186; doi:10.1371/journal.pone.0330989)
Supplement: S8 Table — (PDF) [file pone.0330989.s008.pdf]

Table S8: Robustness check with shop level fixed effects

N=2136	**Primary Outcome**			**Secondary Outcome**			
	**Quantities of all products sold**	**HIV self-test kit sold**	**SRH products sold**	**Condoms sold**	**Emergency Contraception sold**	**Oral contraception sold**	**Pregnancy tests sold**
**Group**							
- No feedback	-	-	-	-	-	-	-
- Private	23<br>(-14, 60)	0.46<br>(-1.40, 2.32)	2.55<br>(-3.97, 9.07)	1.62<br>(-1.05, 4.30)	0.20<br>(-1.12, 1.52)	0.07<br>(-2.34, 2.47)	0.66<br>(-1.00, 2.32)
- Public	25<br>(-24, 74)	-0.20<br>(-2.28, 1.88)	7.65<br>(-3.40, 18.70)	-0.07<br>(-3.91, 3.77)	1.75<br>(-1.13, 4.63)	4.93<br>(-0.06, 9.93)	1.04<br>(-0.82, 2.90)
Outcome mean	194	9.90	16.61	4.81	2.53	4.90	4.37
R2	0.63	0.46	0.70	0.66	0.61	0.69	0.67

*p<0.05, **p<0.01, **p<0.001. Coefficients and 95% confidence intervals in brackets.
