# Supplementary figures and images for: Boosting health provider performance with non-financial incentives: A cluster-randomized controlled trial in Tanzania
Source: PLoS One. 2025 Sep 11;20(9):e0330989. doi: 10.1371/journal.pone.0330989 (PMC12425186; doi:10.1371/journal.pone.0330989)

Figure S9: Heterogeneity by social image dimensions

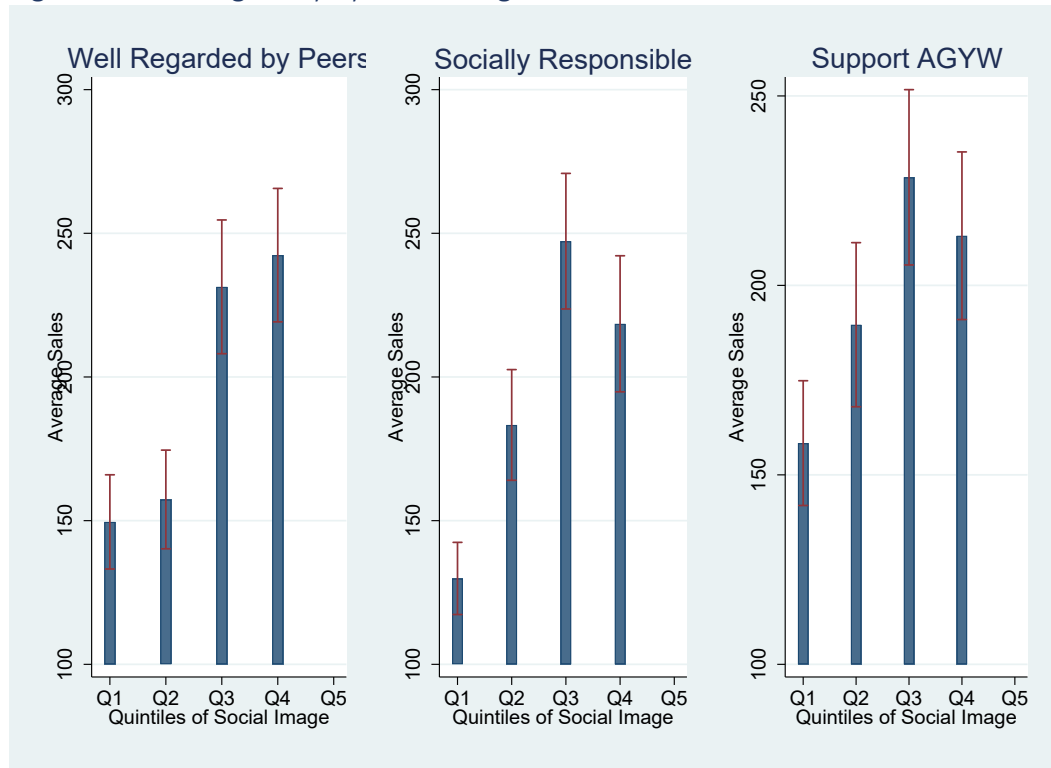

Supplement: S9 Fig — (PDF) [file pone.0330989.s009.pdf]
