# Supplementary material for: Boosting health provider performance with non-financial incentives: A cluster-randomized controlled trial in Tanzania
Source: PLoS One. 2025 Sep 11;20(9):e0330989. doi: 10.1371/journal.pone.0330989 (PMC12425186; doi:10.1371/journal.pone.0330989)
Supplement: S10 Table — (PDF) [file pone.0330989.s010.pdf]

Table S10: Impact of customer feedback on overall HIVST/contraceptives distributed with multiple hypothesis testing corrections

N=2136	**Primary Outcomes**		
	**Quantities of all products sold**	**HIV self-test kit sold**	**SRH products sold**
**Group**			
- No feedback (ref)	-	-	-
- Private	47<br>(-18, 113)<br>p=0.15, BH=0.95	0.17<br>(-1.87, 2.24)<br>p=0.86, BH=1.00	7.58<br>(-2.92, 18)<br>p=0.16, BH=0.93
- Public	113**<br>(38, 189)<br>p=0.004, BH=0.022	1.55<br>(-0.73, 3.82)<br>p=0.17, BH=1.00	16*<br>(0.64, 31)<br>p=0.041, BH=0.245
Outcome mean	194	9.90	16.61
R2	0.06	0.06	0.14

N=2136	**Secondary Outcomes**			
	**Condoms sold**	**Emergency Contraception sold**	**Oral contraception sold**	**Pregnancy tests sold**
**Group**				
- No feedback	-	-	-	-
- Private	3.63<br>(-1.37, 8.63)<br>p=0.15, BH=1.00	1.32<br>(-0.36, 3.01)<br>p=0.12, BH=0.98	0.17<br>(-3.38, 3.72)<br>p=0.93, BH=1.00	2.46<br>(-0.11, 5.02)<br>p=0.060, BH=0.30
- Public	1.50<br>(-1.50, 4.50)<br>P=0.32, BH=1.00	3.38<br>(-0.54, 7.30)<br>p=0.09, BH=0.73	7.57*<br>(0.42, 15)<br>p=0.038, BH=0.30	3.29*<br>(0.33, 6.25)<br>p=0.029, BH=0.237
Outcome mean	4.81	2.53	4.90	4.37
R2	0.07	0.13	0.13	0.13

*p<0.05, **p<0.01, **p<0.001. Coefficients and 95% confidence intervals in brackets. P-values from clustered standard errors and Benjamini-Hochberg adjusted p-values presented.
