# Supplementary material for: Boosting health provider performance with non-financial incentives: A cluster-randomized controlled trial in Tanzania
Source: PLoS One. 2025 Sep 11;20(9):e0330989. doi: 10.1371/journal.pone.0330989 (PMC12425186; doi:10.1371/journal.pone.0330989)
Supplement: S11 Table — (PDF) [file pone.0330989.s011.pdf]

Table S11: Comparison of customer feedback survey response rates by treatment arm

N=582	Customer feedback survey response rate				
	**Mean**	**Median**	**25th percentile**	**75th percentile**	**N**
**Group**					
- Private	0.122	0.024	0.000	0.068	291
- Public	0.125	0.034	0.007	0.093	291

Descriptive statistics computed for customer feedback survey response rate, computed as total number of customer feedback survey responses recorded per month per shop divided by quantities of all products sold per shop per month, by treatment arm (N=582 shop-month observations). P=0.940 in t-tests comparing differences in mean survey response rate by treatment arm.
