# Supplementary material for: Boosting health provider performance with non-financial incentives: A cluster-randomized controlled trial in Tanzania
Source: PLoS One. 2025 Sep 11;20(9):e0330989. doi: 10.1371/journal.pone.0330989 (PMC12425186; doi:10.1371/journal.pone.0330989)
Supplement: S1 File — (PDF) [file pone.0330989.s012.pdf]

TITLE: Supporting Young Women's Reproductive Health by Harnessing Prosociality Among Drug Shopkeepers

INVESTIGATORS AND INSTITUTIONAL AFFILIATIONS:

Werner Maokola, MD, Msc, PhD, National AIDS Control Program
Amon Sabasaba, MD, MSc, Health for a Prosperous Nation
Tracy Kuo Lin, PhD, University of California, San Francisco
Jenny Liu, PhD, University of California, San Francisco

COLLABORATING INSTITUTIONS:

Health for a Prosperous Nation (HPON) is the local not-for-profit agency that will recruit participants and administer the surveys.

Table of Contents

ABBREVIATIONS 3
SUMMARY 3
SPECIFIC AIMS: 4
BACKGROUND: 4
JUSTIFICATION: 4
HYPOTHESIS: 4
GENERAL OBJECTIVES: 5
STUDY DESIGN AND METHODOLOGY: 5
- Overview. 5
- Study populations. 5
- Data collection. 5
- Sampling. 6
- Recruitment Procedures 6
- Data management. 7
- Potential Risk. 8
- Data analysis. 8
- Study Team. 9

TIME FRAME / DURATION OF THE PROJECT 9
EXPECTED APPLICATION OF RESULTS 9
REFERENCES 10

ABBREVIATIONS

AGYW: (Adolescent Girls and Young Women)
CRCT: (Cluster Randomized Control Trial)
HCD: (Human Centered Design)
HIV: (Human Immunodeficiency Virus)
HIVST: (HIV self-testing)
HPON: (Health For a Prosperous Nation)
IRB: (Institutional Review Board)
PrEP: (Pre-exposure Prophylaxis)
SAE: (Serious Adverse Event)
SRH: (Sexual and Reproductive Health)

SUMMARY

In sub-Saharan Africa, adolescent girls and young women (AGYW; ages 15-24) face the dual threats of HIV and unintended pregnancy. Despite an urgent need to reach AGYW with sexual and reproductive health (SRH) services, and the availability of new technologies to support self-care (e.g., HIV self-testing (HIVST) kits, pre-exposure prophylaxis (PrEP)), health systems still fail to get these critical resources to AGYW in need. With the successful introduction of malaria services, Accredited Drug Dispensing Outlets (ADDOs) are already widely recognized as a model for building a lower-skilled cadre of health providers and increasing population-wide access to other basic health services. However, ADDO owners and dispensers may lack motivation to provide high quality of services to AGYW given competing business priorities.

We propose to operationalize and test the preliminary impact of a behavioral economics informed intervention involving providing regular customer feedback from AGYW to ADDO shopkeepers on ADDO service to AGYW customers. This study uses a randomized controlled trial assigning ~N=120 ADDOs to one of three initial conditions: 1. No feedback. 2. Private feedback: Feedback from AGYW customers will be collected anonymously through USSD-code based surveys. Monthly summarized reports of AGYW customer feedback will be given directly to shopkeepers. 3. Public feedback: Shopkeepers will get monthly summarized reports of AGYW customer feedback. In addition, gold stars reflecting the level of AGYW customer feedback (e.g., 1-4 star rating) will be placed in the shop in a visible location, and shopkeepers will be invited to an awards ceremony every 6 months to recognize shopkeepers who receive high levels of positive AGYW customer feedback.

We hypothesize that providing customer feedback to ADDOs will increase provision of services to AGYW. Ex ante, we believe that Private feedback will stimulate ADDO owners and dispensers' underlying pro-social motivation towards supporting AGYW, while Public feedback will additionally introduce social image considerations as ADDOs will want to signal their pro-sociality to the community and compete with other ADDOs. We are agnostic regarding whether Private or Public feedback will be more effective at increasing ADDO performance.

Our study tests the effectiveness of important non-financial incentives on ADDO performance. Should we find evidence of effectiveness, our intervention can be easily scaled across Tanzania to stimulate additional effort among ADDOs in serving AGYW at low cost.

SPECIFIC AIMS:

Aim 1: Operationalize and test the preliminary impact of providing regular AGYW customer feedback to shopkeepers on provision of services to AGYW over 18 months

BACKGROUND:

In sub-Saharan Africa, adolescent girls and young women (AGYW; ages 15-24) face the dual threats of HIV and unintended pregnancy. AGYW comprise 25% of new HIV infections and disproportionately bear 44% of reported unintended births.[1–4] Prevention failures are multi-faceted, including stigma, lack of perceived risk, provider biases, among others.[5–10] Despite an urgent need to reach AGYW with sexual and reproductive health (SRH) services, and the availability of new technologies to support self-care (e.g., HIV self-testing (HIVST) kits, PrEP), health systems still fail get these critical resources to AGYW in need.

Our ultimate goal is to reduce HIV incidence and unintended pregnancies by creating a sustainable, easily accessible, community-based platform for delivering HIV prevention and SRH services to AGYW. Our recent R34MH116804 pilot study in Tanzania showed that Accredited Drug Dispensing Outlets (ADDOs) are effective, community-based spaces for delivering HIVST and contraceptives to AGYW through our Queen Club intervention, developed using human-centered design (HCD) and behavior change tools from behavioral economics.[11] With the successful introduction of malaria services, ADDOs are already widely recognized as a model for building a lower-skilled cadre of health providers and increasing population-wide access to other basic health services. However, ADDO owners and dispensers may lack motivation to provide high quality of services to AGYW given competing business priorities. We propose to operationalize and test the preliminary impact of a behavioral economics informed intervention involving providing regular customer feedback from AGYW to ADDO shopkeepers on ADDO service to AGYW customers.

JUSTIFICATION:

ADDOs are an accessible and sustainable channel to deliver health services to AGYW but are underutilized for HIV prevention. There are over 28,000 new HIV infections among AGYW each year and increased access to services is urgently needed. Aligned with Tanzania's National HIV Strategy for >30% of services to be community-based, ADDOs are common, can distribute HIV self-tests (HIVST), and are often women's first stop for drugs, contraceptives, informal counseling, and health referrals. Our study tests the effectiveness of important non-financial incentives on ADDO performance. Should we find evidence of effectiveness, our intervention can be easily scaled across Tanzania to stimulate additional effort among ADDOs in serving AGYW at low cost.

HYPOTHESIS:

We hypothesize that providing customer feedback to ADDOs will increase provision of services to AGYW. Ex ante, we believe that Private feedback will stimulate ADDO owners and dispensers' underlying pro-social motivation[12] towards supporting AGYW, while Public feedback will

additionally introduce social image[13,14] considerations as ADDOs will want to signal their pro-sociality to the community and compete with other ADDOs. We are agnostic regarding whether Private or Public feedback will be more effective at increasing ADDO performance.

GENERAL OBJECTIVES:

1) To operationalize and test the preliminary impact of a behavioral economics informed interventions involving providing regular customer feedback from AGYW to ADDO shopkeepers

STUDY DESIGN AND METHODOLOGY:

Overview.

We will conduct a randomized controlled trial assigning ~N=120 ADDOs to one of three initial conditions:

1. No feedback.
2. Private feedback: Feedback from AGYW customers will be collected anonymously through USSD-code based surveys. Monthly summarized reports of AGYW customer feedback will be given directly to shopkeepers.
3. Public feedback: Shopkeepers will get monthly summarized reports of AGYW customer feedback. In addition, gold stars reflecting the level of AGYW customer feedback (e.g., 1-4 star rating) will be placed in the shop in a visible location, and shopkeepers will be invited to an awards ceremony every 6 months to recognize shopkeepers who receive high levels of positive AGYW customer feedback.

We will phase in the 2 interventions (Private and Public Feedback) on a staggered basis to control for both shop-specific and time-specific trends for the primary outcome – number of services delivered by ADDOs to AGYW over the 18-month study period. As the gold standard for evaluating the effectiveness of interventions, we will provide robust evidence on the effectiveness of our behavioral economics informed customer feedback intervention on service distribution to AGYW.

Study populations. Our proposed research will recruit ADDOs participating in a parent cluster-randomized trial evaluating the effectiveness of a girl-friendly loyalty program in the Shinyanga and Mwanza regions of Tanzania (NIMR/HQ/R.8a/Vol.IX/3837). In these regions, female HIV prevalence is 7.2% and 8.9%, respectively,[15] only 19.6% of married women use modern contraception, and 34% and 28% of AGYW aged 15-19 have begun childbearing (among the highest in Tanzania), respectively.[16]

Data collection. The primary outcome for this study – the number of services provided by ADDOs to AGYW over the study period – will be collected using electronic sales records maintained by each ADDO using the Maisha Meds inventory management system as part of their participation in the parent trial. As part of the intervention, participating ADDOs will encourage AGYW customers to complete anonymous USSD-based short surveys (see attached for

instrument) regarding their shopping experience at the ADDO. Participation is completely voluntary and at the discretion of the ADDO owner/dispenser (who decides which customers to approach to complete the survey) and the AGYW customer (whose participation in the USSD survey is completely voluntary) with no involvement of the research team beyond setting up the USSD survey infrastructure. This customer feedback data will be summarized into digestible reports and provided to ADDOs monthly to encourage them to increase service provision to AGYW and highlight areas for improvement. Among ADDOs assigned to the Public feedback arm, the research team will additionally display gold stars reflecting the level of AGYW customer feedback (e.g., 1-4 star rating) in a visible location and invite ADDO owners and dispensers to an awards ceremony every 6 months to recognize shopkeepers who receive high levels of positive AGYW customer feedback.

ADDOs will be assigned to one of three initial conditions:

1. No feedback.
2. Private feedback
3. Public feedback

We will phase in the 2 interventions (Private and Public Feedback) on a staggered basis (Figure 1) to control for both shop-specific and time-specific trends for the primary outcome – number of services delivered by ADDOs to AGYW over the 18-month study period.

R03 study arm	Phase 0: Pre-intervention	Phase 1: Initial condition	Phase 2: Added condition
	0-6 months	***6-12 months***	***12-18 months***
Group 1 (N=40)	No feedback	No feedback	**Private** feedback
Group 2 (N=40)	No feedback	**Private** feedback	**Public** feedback
Group 3 (N=40)	No feedback	**Public** feedback	**Public** feedback

Figure 1. Treatment assignments for different shops over the study period. The private public feedback arms are phased-in across different shops on a staggered basis to allow us to analytically control for both shop- and time-specific trends.

Sampling. All ADDOs participating in the parent trial (~N=120) will be recruited to participate in this study. The sample is a convenience sample but gives us power to detect a 20% relative increase between Private Feedback and No Feedback, and Public Feedback and No Feedback respectively. Minimum detectable effect sizes vary between 15-19%, depending on the treatment arm considered and the outcome. Additionally, we are powered to detect a 15% relative increase between the pooled Private and Public Feedback arms and No Feedback. The magnitudes of these effects are plausible and consistent with estimates from similar interventions in the Behavioral Economics literature[17]. We will potentially not be powered to detect differences between Private and Public Feedback if effect differences are small. Similarly, we will not be powered to detect differences in the treatment effect by the parent trial treatment arms; thus, we focus our analysis on estimating main effects of the proposed interventions. Given the reliance on routinely collected data from shops' electronic inventory management system to measure the primary outcomes, attrition is not applicable.

Recruitment Procedures. All ADDOs participating in the parent trial will be invited to participate in this ancillary study testing the impact of receiving regular AGYW customer feedback on the provision of services to AGYW. Those who are interested in participating will be asked to provide written informed consent agreeing to elicit AGYW customer feedback through anonymous

USSD-based short surveys, receive summary reports monthly, and publicly display performance scores if randomized to the Public Feedback arm. There are no exclusion criteria.

Upon first contact with a potential participant for the survey, research staff will orally inform the potential participant about the purpose and nature of the study. Research staff will then complete the informed consent process with individuals who are interested in participating in the study in a quiet and private area. As part of the informed consent process, the research staff will explain the study and its goals and review all of the elements of informed consent with the individual. At this point, potential participants will be assured that refusing participation will not impact them in any way, and that participation is entirely voluntary. In addition, individuals will be reminded that they can refuse to answer any question or leave the interview/discussion at any time.

After discussing the consent form with study staff, participants will be quizzed about the nature of participation to ensure that they have adequately understood the information. (Note: Consent forms will be read aloud to potential participants unless individuals insist that they are comfortable reading the form themselves). Individuals who would like to participate can sign the informed consent form, or, for participants who feel uncomfortable signing the consent form, they will be asked to put a thumbprint (instead of a signature) at the bottom of the document after the interviewer has fully read, explained, and answered questions related to the consent form and study participation in general. In addition, study staff will sign the two copies of the consent form, noting that they have obtained written informed consent. A copy of the signed informed consent form will be provided to participants who wish to have a copy.

Data management. Our research team has considerable institutional expertise and well-established systems for data collection and management. In addition, H-PON has established a research site and has hired and trained study personnel in Mwanza and Shinyanga who also adhere to these systems. These staff have successfully conducted human subjects research under the direction of the PI, and local collaborators Dr. Sabasaba (HPON) on four previous studies (including the pilot randomized trial for the parent study and the parent CRCT) and two current ongoing studies in the same area. They have undergone training on obtaining informed consent, including for the participation of minors, in each of these other projects. A refresher training will also be conducted as part of this study in order to ensure that study specific information and procedures are followed with fidelity.

A key policy of our collaborative research group is that the fewer individuals handling sensitive information, the greater the protection. Thus, project files and databases will only be available to research personnel through the authorization of the PI. All staff with access to the data or who interact with participants must sign confidentiality agreements and will be informed of their responsibility to maintain confidentiality once they have left the study or the study has ended. Staff will be trained on the policies and procedures for data management and transmission and will also receive instructions on how to report any violations of those policies and procedures. All staff will be appropriately trained in the handling of sensitive study data through their employment training and annual completion of training in the ethical conduct of research.

The PI will review all procedures for protecting confidentiality with study staff on a bi-monthly basis, including storing the data, consents, and questionnaires in secure databases and locked file

cabinets in locked offices, password protection, and procedures for transferring study data to the secure password-protected cloud-based folder. In addition, the team has a standard operating procedure in place to conduct quality control spot checks and ensure the privacy and confidentiality of study participants, data collection forms, and electronic data.

Potential Risk. There are no physical risks associated with this study. This research presents no risks of harm greater than the probability and magnitude encountered in daily life. The study involves i) asking ADDO owners/dispensers to ask AGYW customers to fill out a quick, anonymous USSD survey, ii) receiving summary reports on these surveys, and iii) publicly displaying feedback information and participating in awards ceremonies if assigned to the Public feedback arm. No personal identifying information will be collected from ADDO owners or dispensers. The primary outcomes involve administrative data on sales and services provided to AGYW, which is not sensitive.

We believe that the benefits of the study warrant the relatively low risks posed to subjects in the study. The results from the study will add to generalizable knowledge about how to increase services delivered to AGYW through ADDOs. There are over 28,000 new HIV infections among AGYW each year and increased access to services is urgently needed. Aligned with Tanzania's National HIV Strategy for >30% of services to be community-based, ADDOs are common, can distribute HIV self-tests (HIVST), and are often women's first stop for drugs, contraceptives, informal counseling, and health referrals. Our study tests the effectiveness of important non-financial incentives on ADDO performance. Should we find evidence of effectiveness, our intervention can be easily scaled across Tanzania to stimulate additional effort among ADDOs in serving AGYW at low cost.

All participants will be informed that all data collection activities are confidential (as stated in the informed consent process) prior to their agreement to participate. There will be no identifiable individual information collected as part of this study. Only study investigators and study personnel will have access to the study files. **Serious Adverse Events.** As all procedures in this study are standard survey procedures recommended by the Society of Medical Decision Making and UCSF Institutional Review Board. The risks to participation in general are minimal, and this study will not monitor or collect data on adverse events. In the unlikely event that a serious adverse event (SAE) is considered possibly, probably or definitely related to the study, or in the event of unexpected incidents or protocol violations, reporting to UCSF and NIMR IRBs will be made. Suspected events will be reviewed by study investigators prior to submission to the IRBs.

Data analysis. Our study team has considerable institutional expertise and well-established systems for data collection and management. In addition, our partner in Tanzania, HPON, has established a research site and has hired and trained study personnel in Shinyanga and Mwanza who also adhere to these systems. A key policy of our collaborative research group is that the fewer individuals handling sensitive information, the greater the protection. Thus, project files and databases will only be available to research personnel through the authorization of the PI (Dr. Liu). All staff with access to the data or who interact with participants must sign confidentiality agreements and will be informed of their responsibility to maintain confidentiality once they have left the study or the study has ended. Staff will be trained on the policies and procedures for data management and transmission and will also receive instructions on how to report any violations

of those policies and procedures. All staff will be appropriately trained in the handling of sensitive study data through their employment training and annual completion of training in the ethical conduct of research. The PI will review all procedures for protecting confidentiality with study staff on a bi-monthly basis, including storing the data, informed consent and assent forms, and questionnaires in secure databases and locked file cabinets in locked offices, password protection, and procedures for transferring study data to the secure password-protected cloud-based folder. In addition, the team has a standard operating procedure in place to conduct quality control spot checks and ensure the privacy and confidentiality of study participants, data collection forms, and electronic data.

Sales data will be aggregated in monthly increments, mirroring shopkeepers' receipt of customer feedback in intervention groups. We will estimate generalized linear regressions controlling for shop- (γi) and time- (dt) specific trends, and parent trial study arm (Mi): $Yi,t = \alpha + \beta 1Ti,t + \gamma i + Mi + dt + \varepsilon i,t$, where Yi,t is the outcome of interest by shop i in time period t. $\beta 1$ represents the mean change in the outcome between shops receiving Private or Public feedback (Tit) relative to No feedback

Study Team. Dr. Werner Maokola will oversee all aspects of recruitment and data collection. Drs. Amon Sabasaba and Jenny Liu will oversee data analysis. All investigators will be involved in manuscript preparation and dissemination of results.

TIME FRAME / DURATION OF THE PROJECT

The study will take place over 18 months. Private and Public feedback will be phased in among different shops over three 6-month study phases. The study will recruit ADDOs participating in an ongoing cluster-randomized trial of a girl-friendly loyalty program in the Shinyanga and Mwanza regions of Tanzania (NIMR/HQ/R.8a/Vol.IX/3837). Data analysis will take place in both Shinyanga and Mwanza regions of Tanzania, and in California, United States.

EXPECTED APPLICATION OF RESULTS

ADDOs are an accessible and sustainable channel to deliver health services to AGYW but are underutilized for HIV prevention. There are over 28,000 new HIV infections among AGYW each year and increased access to services is urgently needed. Aligned with Tanzania's National HIV Strategy for >30% of services to be community-based, ADDOs are common, can distribute HIV self-tests (HIVST), and are often women's first stop for drugs, contraceptives, informal counseling, and health referrals. Our study tests the effectiveness of important non-financial incentives on ADDO performance. Should we find evidence of effectiveness, our intervention can be easily scaled across Tanzania to stimulate additional effort among ADDOs in serving AGYW at low cost. Results will be disseminated to relevant stakeholders at the Ministry of Health.

REFERENCES

1. Fleischman J, Peck K. Addressing HIV Risk in Adolescent Girls and Young Women [Internet]. Washington, D.C.: Center for Strategic & International Studies; 2015 Apr. Available from: https://csis-prod.s3.amazonaws.com/s3fs-public/legacy_files/files/publication/150410_Fleischman_HIVAdolescentGirls_Web.pdf

2. UNAIDS. HIV Prevention among Adolscent Girls and Young Women [Internet]. Geneva, Switzerland: Joint United Nations Programme on HIV/AIDS (UNAIDS); 2016. Available from: http://www.unaids.org/sites/default/files/media_asset/UNAIDS_HIV_prevention_among_adolescent_girls_and_young_women.pdf

3. UNAIDS, The African Union. Empower Young Women and Adolescent Girls: Fast-tracking the End of the AIDS Epidemic in Africa [Internet]. Joint United Nations Programme on HIV/AIDS (UNAIDS); 2015. Report No.: JC2746. Available from: http://www.unaids.org/sites/default/files/media_asset/JC2746_en.pdf

4. Hubacher D, Mavranezouli I, McGinn E. Unintended pregnancy in sub-Saharan Africa: magnitude of the problem and potential role of contraceptive implants to alleviate it. Contraception. 2008 Jul;78(1):73–78. PMID: 18555821

5. Napierala Mavedzenge SM, Doyle AM, Ross DA. HIV Prevention in Young People in Sub-Saharan Africa: A Systematic Review. J Adolesc Health. 2011 Dec;49(6):568–586. PMID: 22098767

6. Michielsen K, Chersich MF, Luchters S, De Koker P, Van Rossem R, Temmerman M. Effectiveness of HIV prevention for youth in sub-Saharan Africa: systematic review and meta-analysis of randomized and nonrandomized trials. Aids. 2010;24(8):1193–1202. PMID: 20375876

7. McCoy SI, Kangwende RA, Padian NS. Behavior Change Interventions to Prevent HIV Infection among Women Living in Low and Middle Income Countries: A Systematic Review. AIDS Behav. 2009 Dec 1;14(3):469–482. PMID: 19949847

8. McCoy SI, Ralph LJ, Njau PF, Msolla MM, Padian NS. Food Insecurity, Socioeconomic Status, and HIV-Related Risk Behavior Among Women in Farming Households in Tanzania. AIDS Behav. 2013 Oct 6;18(7):1224–1236. PMID: 24097335; PMCID: PMC3977025

9. Dunbar MS, Kang Dufour MS, Lambdin B, Mudekunye-Mahaka I, Nhamo D, Padian NS. The SHAZ! project: results from a pilot randomized trial of a structural intervention to prevent HIV among adolescent women in Zimbabwe. PloS One. 2014;9(11):e113621. PMID: 25415455; PMCID: PMC4240618

10. Dunbar MS, Maternowska MC, Kang MSJ, Laver SM, Mudekunye-Mahaka I, Padian NS. Findings from SHAZ!: a feasibility study of a microcredit and life-skills HIV prevention

intervention to reduce risk among adolescent female orphans in Zimbabwe. J Prev Interv Community. 2010;38(2):147–161. PMID: 20391061; PMCID: PMC4578719

11. Hunter LA, McCoy SI, Rao A, Mnyippembe A, Hassan K, Njau P, Mfaume R, Liu JX. Designing drug shops for young women in Tanzania: applying human-centred design to facilitate access to HIV self-testing and contraception. Health Policy Plan. Oxford Academic; 2021 Nov 11;36(10):1562–1573.

12. Besley T, Ghatak M. Prosocial Motivation and Incentives. Annu Rev Econ. 2018;10(1):411–438.

13. Bursztyn L, Jensen R. Social Image and Economic Behavior in the Field: Identifying, Understanding, and Shaping Social Pressure. Annu Rev Econ. 2017;9(1):131–153.

14. Ashraf N, Bandiera O. Social incentives in organizations. Annu Rev Econ. Annual Reviews; 2018;10:439–463.

15. Tanzania Commission for, Zanzibar AIDS Commission AC. Tanzania HIV Impact Survey (THIS) 2016-2017: Final Report. Dar es Salaam, Tanzania; 2018 Dec.

16. Ministry of Health, Community Development, Gender, Elderly and Children (MoHCDGEC) [Tanzania Mainland], Ministry of Health (MoH) [Zanzibar], National Bureau of Statistics (NBS), Office of the Chief Government Statistician (OCGS), ICF International. Tanzania Demographic and Health Survey and Malaria Indicator Survey (TDHS-MIS) 2015-16 [Internet]. Dar es Salaam, Tanzania, and Rockville, Maryland, USA: MoHSW, MoH, OCGS, and ICF International; 2016. Report No.: PR74. Available from: http://dhsprogram.com/pubs/pdf/PR74/PR74.pdf

17. DellaVigna S, Linos E. RCTs to Scale: Comprehensive Evidence from Two Nudge Units [Internet]. Cambridge, MA: National Bureau of Economic Research; 2020 Jul p. w27594. Report No.: w27594. Available from: http://www.nber.org/papers/w27594.pdf
