# Supplementary material for: Boosting health provider performance with non-financial incentives: A cluster-randomized controlled trial in Tanzania
Source: PLoS One. 2025 Sep 11;20(9):e0330989. doi: 10.1371/journal.pone.0330989 (PMC12425186; doi:10.1371/journal.pone.0330989)
Supplement: S2 File — (PDF) [file pone.0330989.s013.pdf]

Inclusivity in global research

PLOS' policy on inclusivity in global research aims to improve transparency in the reporting of research performed outside of researchers' own country or community and ensures that PLOS publications reporting global research adhere to high standards for research ethics and authorship. Authors of relevant research articles may be asked to complete the questionnaire below, which outlines ethical, cultural, and scientific considerations specific to inclusivity in global research. This questionnaire may be requested when researchers have travelled to a different country to conduct research, if research uses samples collected in another country, research with Indigenous populations or their lands, or if research is on cultural artefacts. Researchers travelling to another country solely to use laboratory equipment will not normally be required to complete the questionnaire. However, the questionnaire can be requested at the journal's discretion for any submission – if you have been requested to complete this questionnaire by the PLOS journal you submitted to, please do so.

Please complete the questionnaire below and include this as a Supporting Information file with your manuscript. Note that if your paper is accepted for publication, this checklist will be published with your article in the supporting information files. Please ensure that you reference the checklist in the main body of your manuscript. We suggest adding a subsection 'Inclusivity in global research' to your Methods section and adding the following sentence: "Additional information regarding the ethical, cultural, and scientific considerations specific to inclusivity in global research is included in the Supporting Information (SX Checklist)"

The questions have been designed to be applicable to a wide range of study types, and there are subsections for both human subjects research and non-human subjects research. If any of the questions are not relevant to your research please mark them as "N/A" as appropriate.

Ethical considerations, permits and authorship

This section is applicable to all research types.

Provide details as to who granted permissions and/or consent for the study to take place in the Methods section of your manuscript. This should include the names of **all** ethics boards, governmental organizations, community leaders or other bodies that provided approval for the study. If individuals provided approval refer to these people by their role or title but do not list their name(s).

Reported on page number: 6

If there were any deviations from the study protocol after approval was obtained please provide details of these changes in the Methods section of your manuscript.

Reported on page number: 9, 10

Did this study involve local collaborators that are residents of the country where the research was conducted or members of the community studied? If you do not have any authors from said communities, please provide an explanation for this below.

Yes, the study involved local collaborators that are residents of Tanzania. Co-authors Agatha Mnyippembe and Emmanuel Katabaro from Health For a Prosperous Nation participated in the conception and design of the study and led all data collection and implementation efforts. Co-author Werner Maokola from the Ministry of Health participated in the study design and manuscript preparation, and provided input on the study design and human subjects review to ensure that the intervention was appropriate for the study population and also reflected policy priorities from the Ministry of Health.

Everyone listed as an author should meet PLOS' criteria for authorship and all individuals who meet these criteria should be included in the author byline, rather than the acknowledgements. For further information please see the journal's Authorship Policy.

Human subjects research (e.g. health research, medical research, cross-cultural psychology)

Did you obtain written informed consent from a representative of the local community or region before the research took place? How did you establish who speaks for the community? Details of written informed consent obtained from study participants should be reported separately in the Methods section of your manuscript.

No, we did not obtain written informed consent from a representative of the local community before research took place. However, we conducted extensive engagement with the local community in preparation for the study, including regular meetings with local and regional government officials such as representatives of the regional pharmacy council. The study was conceived and co-designed in collaboration with local government officials who provided letters of support at the grant application stage. Further, we engaged with the Youth Advisory Board (YAB) from the parent study to discuss the design of this study, including the piloting and refining of intervention procedures based on their feedback.

We obtained written informed consent from study participants as reported in the Methods section of our manuscript.

How did members of the local community provide input on the aims of the research investigation, its methodology, and its anticipated outcome(s)?

We engaged with the YAB from the parent study to design the customer feedback survey – we revised the survey length, content, and format based on their input to ensure high degree of acceptability of the questions and smooth implementation. We also engaged with drug shops in the local community to provide input on the intervention, including the structure and consent of the summary feedback reports. We engaged with drug shops that participated in a previous pilot study who understand the local context but were non-participants of the current study to avoid contamination. All engagement with the local community was conducted by our Tanzania-based research team, led by Agatha Mnyippembe and Emmanuel Katabaro who are co-authors of this study. The research team has long-standing relationships with the local community in the study region, having conducted research in the area for over a decade. The research team regularly interact with the local community and solicit feedback on research activities.

When engaging with the local community, how did you ensure that the informed consent documents and other materials could be understood by local stakeholders?

All engagement with the local community was conducted by our Tanzania-based research team, by native, Swahili-speaking Tanzanians. All informed consent documents for study participants were translated, backtranslated and piloted before use to ensure understanding by study participants.

Will the findings of the research be made available in an understandable format to stakeholders in the community where the study was conducted (e.g. via a presentation, summary report, copies of publications, etc.)? Please provide details of how this will be achieved.

Yes, research findings will be disseminated with regional and national level government officials through in-person dissemination events with presentations from the research team. We will invite representatives from drug shops, including the study participants, to attend regional meetings. In addition, we will disseminate results to the YAB of the parent study.

Non-human subjects research using specimens/ animals collected as part of the study, or those housed in archival collections. Examples include archaeology, paleontology, botany and zoology.

Did the permission you obtained from a local authority to perform the study include an agreement on access to outputs and benefit sharing? This may include procedures to enable fair distribution of the benefits and resources arising from the research performed. Please include any details of Prior Informed Consent and Benefit Sharing Agreements obtained. These may be required by field-specific regulations, for example the Convention on Biological Diversity (CBD) and the associated Nagoya Protocol.

N/A

If the material used in your study was imported, please A) provide the year it was imported and B) indicate whether permits were obtained to import/export the materials used, C) provide details of any permits obtained. If this information is not available, please indicate this.

N/A

If you used archival specimens, please state how the material used in your study was acquired by the institute it is held in and provide details of any permits obtained for the original excavations/ sample collection. If this information is not available, please indicate this.

N/AN

How was the potential cultural significance of the materials collected in your study to local communities considered in your research design? Were Indigenous peoples and/or local researchers and institutions involved with archaeological excavations / collection of specimens? If so, please provide a description of their involvement.

N/A

If your manuscript includes photographs of human remains please indicate whether authors obtained permission from descendants or affiliated cultural communities to do so.

N/A
